# A Systematic Review and Meta-Analysis of the Impact of Different Intensity of Dietary Counselling on Cardiometabolic Health in Middle-Aged and Older Adults

**DOI:** 10.3390/nu13092936

**Published:** 2021-08-25

**Authors:** Jasmine Hui Min Low, Darel Wee Kiat Toh, Magdeline Tao Tao Ng, Johnson Fam, Ee Heok Kua, Jung Eun Kim

**Affiliations:** 1 Department of Food Science & Technology, Faculty of Science, National University of Singapore, Singapore 117543, Singapore; jasmine.low@u.nus.edu (J.H.M.L.); dareltoh@u.nus.edu (D.W.K.T.); 2 National University of Singapore Libraries, National University of Singapore, Singapore 117543, Singapore; magdeline.ng@nus.edu.sg; 3 Department of Psychological Medicine, National University of Singapore, Singapore 119228, Singapore; pcmjf@nus.edu.sg (J.F.); pcmkeh@nus.edu.sg (E.H.K.)

**Keywords:** nutritional strategy, nutritional counselling, cardiometabolic risk, older adults

## Abstract

Dietary counselling has been identified as one of the nutritional strategies to alleviate cardiometabolic health conditions. Its effectiveness however may vary due to factors such as intensity level and provider while this has not been comprehensively studied. This systematic review and meta-analysis aimed to assess the effects of dietary counselling on the cardiometabolic health in middle-aged and older adults and the sub-group analyses with dietary counselling intensity and the provider were also assessed. Four databases including PubMed, CINAHL Plus with Full Text, Cochrane Library and EMBASE were systematically searched. Data from 22 randomised controlled trials (RCTs) were compiled and those from 9 RCTs were utilised for meta-analysis. Dietary counselling lowered total cholesterol (TC) and fasting blood sugar (FBS) but had no impact on triglycerides (TG) and low-density lipoprotein (LDL). Sub-group analysis revealed significant lowering effect of high intensity dietary counselling for TG (weighted mean difference (WMD): −0.24 mmol/L, 95% confidence intervals (CIs): −0.40 to −0.09), TC (WMD: −0.31 mmol/L, 95% CIs: −0.49 to −0.13), LDL (WMD: −0.39 mmol/L, 95% CIs: −0.61 to −0.16) and FBS (WMD: −0.69 mmol/L, 95% CIs: −0.99 to −0.40) while medium or low intensity dietary counselling did not show favouring effects. Counselling provider showed differential responses on cardiometabolic health between dietitian and all other groups. The findings from this systematic review and meta-analysis suggest that dietary counselling is a beneficial dietary strategy to improve cardiometabolic health in middle-aged and older adults with the emphasis on the counselling intensity.

## 1. Introduction

Aging has become a worldwide phenomenon and according to the United Nations, the projected number of older people over the next thirty years will double, reaching over 1.5 billion persons [1]. The largest increase is anticipated to occur in Eastern and South-Eastern Asia [1]. Aging can be associated with increased morbidity including cardiometabolic risks [2]. A longitudinal cohort study reported an increase in cardiometabolic multimorbidity with age from 5.2% and 11.6% in the population aged ≥40 and ≥60 respectively [3].

Some studies have advanced the research in examining the relationship between overall diet quality and cardiometabolic disorders. A wide array of evidence has shown that a lower dietary quality in older age is associated with an increased risk of cardiovascular disease (CVD) and type 2 diabetes mellitus [4,5]. In a recent study conducted to assess the prospective association between a 6-year change in diet quality and the risk for incident CVD, a higher dietary quality was strongly associated with lower CVD risk within the middle-aged community [6]. These findings indicate that diet quality may have a pivotal role in influencing the development and progression of cardiovascular disorders.

Two primary strategies for the management of cardiometabolic risk has been identified such as the modification of underlying risk factors and an isolated treatment for each underlying risk factor [7]. Some of the interventions involved lifestyle change such as altering dietary habit or exercise training and medical procedures such as pharmacologic therapy or weight loss surgical procedure have shown improvements in the management of cardiometabolic risk factors [8,9,10]. However, pharmacological and surgical management may not provide a long-term solution as many other cardiometabolic risk factors may co-occur leading to polypharmacy [11]. Therefore, the introduction of lifestyle changes such as dietary modification and exercise training may be able to provide a long-term and less invasive solution. An example of dietary modification can be achieved through the provision of dietary counselling. Dietary counselling is defined as a two-way interaction between a patient or a group and a member of the medical team through nutritional assessment to identify any nutritional problems, needs, and goals [12]. This is also in accordance with the American Dietetic Association (ADA)’s definition of nutrition counselling as “advising and assisting individuals and groups on appropriate nutrition intake by integrating information from the nutrition assessment with information on food and other sources of nutrients and meal preparation consistent with cultural background and socioeconomic status” [13]. Dietary counselling is suggested to be an effective nutritional strategy to improve the dietary quality in older adults and may prevent the onset of various aged-related health conditions or diseases [14,15]. A recent systematic review and meta-analysis analysing the effectiveness of dietary counselling for lowering blood lipid levels in high-risk individuals observed a significant reduction in triglycerides (TG) levels [16]. Another meta-analysis which assessed the impact of nutrition counselling in diabetic patients observed significant reductions in fasting blood sugar (FBS), total cholesterol (TC) and systolic blood pressure (SBP) [17]. However, a randomised controlled trial (RCT) carried out in Hong Kong which sought to investigate the impact of Dietary Approach to Stop Hypertension (DASH)-based dietary counselling on grade 1 hypertensive patients found no significant reduction in blood pressure over a period of 12 months [18].

Collectively, mixed results of dietary counselling on cardiometabolic health have been observed. Different studies presented different methodological combination of dietary counselling such as the number of times the counselling sessions were provided, how long each session was held and the personnel who conducted the counselling session. A review study conducted by Lin et al. [19] discovered that high intensity dietary counselling trials have shown greater dietary compliance as compared to low and medium intensity trials. Hence, the intensity of dietary counselling has become an important subject for discussion. Moreover, at present, no systematic review has been conducted to examine the effect and the extensivity of dietary counselling on the cardiometabolic health of middle-aged and older adults. Therefore, the aim of this systematic review and meta-analysis is to assess the effects of dietary counselling on cardiometabolic health in the middle-aged and older population. Further subgroup analysis will be performed to elucidate the effect of specific components of dietary counselling (e.g., intensity of dietary counselling and counselling provider) on the cardiometabolic health).

## 2. Materials and Methods 

### 2.1. Registration

This systematic review was completed in adherence to the Preferred Reporting Items for Systematic Reviews and Meta-Analyses (PRISMA) statement [20]. The PICOS (participant, intervention, comparison, outcome, and study design) criteria and the research question is described in Table 1. The review is registered with PROSPERO International prospective register of systematic reviews (CRD4202015379).

### 2.2. Search Strategy and Inclusion Criteria

The article search was conducted by the primary (JHML) and secondary (DWKT) reviewers. A total of four online databases including PubMed, CINAHL Plus with Full Text, Cochrane Library and EMBASE were searched from inception until February 2020 and the search was updated as of April 2021. The key search string used was common across all databases and included (‘diet counseling’ OR ‘dietary counseling’ OR ‘nutritional counseling’ OR ‘nutrition counseling’) AND (‘blood glucose’ OR ‘insulin’ OR ‘blood pressure’ OR ‘lipoprotein, hdl’ OR ‘lipoprotein, ldl’, OR ‘triglycerides’ OR ‘cholesterol’). Human and English language were selected as limiters and medical subject headings (MeSH) used when applicable. Reference lists of relevant reviews were also hand-searched to identify additional articles.

The articles were accepted based on the following search criteria: (1) RCT study design; (2) subject’s mean age ≥50 years old; (3) reporting of cardiometabolic health related classical outcomes [i.e., TC, low density lipoprotein cholesterol (LDL), high density lipoprotein cholesterol (HDL), TG, SBP, diastolic blood pressure (DBP), FBG, and insulin]. Articles that included other life-style interventions such as exercises or the co-consumption of supplementation were excluded. Studies with no intervention control groups or standard care groups were included.

### 2.3. Articles Selection and Data Extraction 

The titles and abstracts of all articles were first screened by the primary and secondary reviewers and disagreements between reviewers were resolved by consensus with a third reviewer (JEK). Articles were rejected after full text screening based on 1 of the 3 reasons: (1) study design was not an RCT, or the intervention included exercises or co-consumption of other supplementation or medication; (2) population was not human or had a mean age of <50 years; (3) outcomes of interest were not analysed. A hand search of relevant reviews from relevant reviews and research articles was also conducted. The following data were extracted from selected articles onto an electronic form: first author name; publication year; population size; drop-out rate; study design and duration; population mean age, body mass index and gender ratio; intervention specifics, including the method of dietary counselling, who conducted the counselling and whether or not additional dietary changes were introduced; method of diet administration; compliance assessment; intensity of dietary counselling; mean and standard deviations (SDs) of the pre-intervention, post-intervention, and change values for selected outcomes variables; and key conclusions. Units for the lipid-lipoproteins were standardized to mmol/L (TC, LDL-C, and HDL-C—mg/dL × 0.02586; TG—mg/dL × 0.01129; FBG—mg/dL × 0.0555). Upon circumstances where clarification was required to obtain unpublished useful data, corresponding authors were contacted via email. Studies that included more than one relevant intervention and control arm were considered as a distinct group for multiple pairwise comparisons to account for within-study variations [21].

### 2.4. Risk of Bias Assessment

The Cochrane Collaboration modified tool for assessing risk of bias for studies was used to determine the quality of the studies selected [22]. A judgement level (high, low, or unclear) was assigned to each article to determine any prevalence of selection bias (random sequence generation, allocation concealment), reporting bias (selective reporting), performance bias (blinding of participants and investigator), detection bias (blinding of outcome assessor) and attrition bias (incomplete outcome data) and other sources of bias.

### 2.5. Calculation and Statistical Analysis

If the SDs of the change values for both the control and intervention groups of each study were unavailable, the values were calculated using a correlation factor representative of the change-value SDs that were available from the other studies [23]. In addition, the post values, the reported and/or calculated change values and their ranges for each outcome were collected.

Obtained data were analysed using STATA (Version 13, StataCorp LP, College Station, TX, USA) for the meta-analysis. The metan function was utilized for the determination of pooled outcome effects. The overall effect sizes for all outcomes were determined using the weighted mean difference (WMD) of the change values between the dietary counselling and no dietary counselling groups with 95% confidence intervals (CIs). Random-effect model was utilized to account for the heterogeneity since variation of dietary counselling within the intervention groups was evident. Positive effect sizes did not favour dietary counselling while negative effect sizes favoured dietary counselling. Heterogeneity was quantified through the Cochran’s Q and I2 statistic, which was derived from chi-square statistic, with a value of more than 50% and a *p*-value < 0.05 indicative of significant heterogeneity [24].

Sub-group analyses were conducted based on the intensity of the dietary counselling and the provider of dietary counselling. With regards to the classification of the dietary counselling intensity, a rating of ‘low’, ‘medium’ or ‘high’ was allocated for each available intervention arm. This was derived based on the number and length of counselling contacts. Low intensity interventions involved 1 contact lasting less than 30 min, high intensity interventions involved contacts greater than 6, each lasting at least 30 min. Dietary counselling intensities in between belonged to medium intensity interventions [25]. For the sub-group analysis according to the provider, it was spilt into dietitian involvement and all others. Any studies that employed the usage of dietitian was grouped together under ‘Dietitian Involvement’ and the rest was classified under ‘All Others’. All others included nutritionist, physician, research staff and nurses. The sensitivity of the result was tested by repeating the meta-analysis with the removal of single pairwise comparisons.

## 3. Results

### 3.1. Study Selection and Subject Characteristics

**Figure 1:**
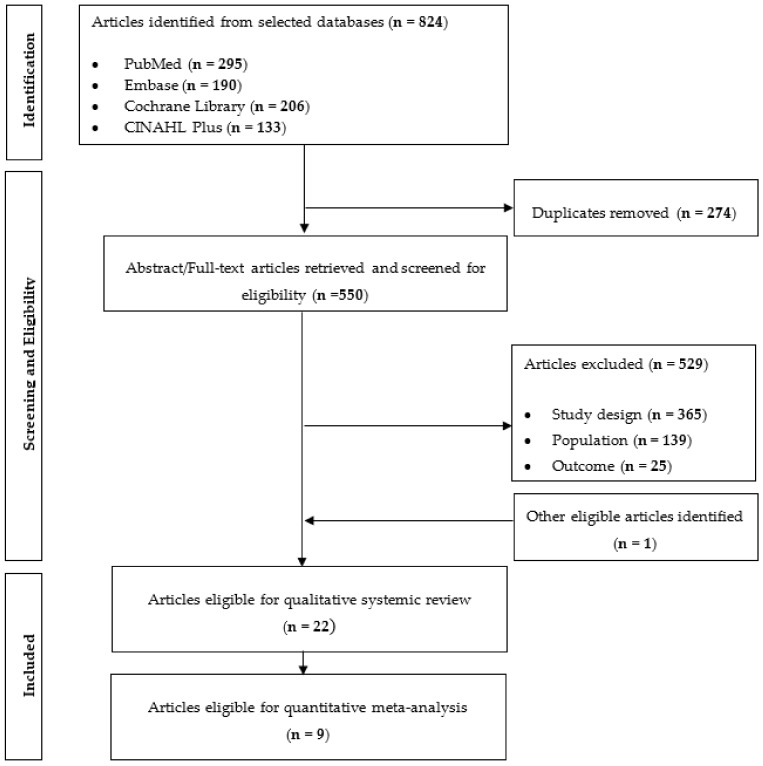
Flow diagram of article search and screening process showing the number of studies assessed for eligibility and included in the review for cardiometabolic health related clinical trials.

A total of 824 articles were obtained through the search and all the identified articles were exported to EndNote X7 (Thomas Research Software, Carlsbad, CA, USA) for article management and the exclusion of duplicates (*n* = 274). Articles were rejected after full text screening based on one of the three reasons: (1) study design was not an RCT, or the intervention included exercises or co-consumption of other supplementation or medication; (2) population was not human or had a mean age of <50 years; (3) outcomes of interest were not analysed. A hand search of relevant reviews and article references yielded one additional article. Bibliographies from relevant reviews and research articles identified were further inspected to obtain 1 additional article for a complete study listing. In total, 22 articles were selected for qualitative systematic review and 9 articles were eligible for quantitative analysis as the remaining 13 articles did not provide sufficient details required for the analysis (Figure 1). All the 22 studies were eligible for cardiometabolic health outcomes [23,26,27,28,29,30,31,32,33,34,35,36,37,38,39,40,41,42,43,44,45,46] and the study features and subject characteristics of the selected articles are summarized in Table 2. A parallel study design was performed in all 22 studies. Amongst the 22 studies, only one of the studies featured generally healthy subjects [43], the remaining of the studies featured subjects with various disease conditions such as type 2 diabetes, hypercholesterolemia, hypertension, metabolic syndrome, certain types of cancer and renal disease.

### 3.2. Risk of Bias Assessment

The risk of bias assessment results are summarized in Supplementary Table S1. Selection bias (random sequence allocation and allocation concealment) was marked ‘low’ for about one-third of the articles, while most were labelled under the unclear category due to the lack of clear description of how the randomization or concealment was conducted. A ‘high’ score was allocated to Gans et al. [36] for random sequence allocation. According to Gans et al. [36], the study site was randomised however the subjects were all exposed to the same intervention. Other sources of bias were marked ‘unclear’ for all the articles. Performance bias and detection bias were marked “unclear” for some of the articles, with a few scored ‘low’ as evident blinding for the respective components were mentioned. Performance bias was marked high for Henkin et al. [37], Muchiri et al. [40], Noda et al. [41], Takahashi et al. [43], Tan et al. [47], Pimentel et al. [45], Klein et al. [29], Cheng et al. [30], Samuelsson et al. [32], Wu et al. [33], and Holland et al. [48]. This was due to the inevitable nature of dietary counselling; subjects were unable to be blinded when undergoing the intervention and hence resulting in only single blinding. Blinding of assessor was marked high for Muchiri et al. [40], Britton et al. [49], Cheng et al. [30], Samuelsson et al. [32], Wu et al. [33], and Holland et al. [48]. Attrition bias was deemed ‘unclear’ for most articles due to the lack of more explicit stating, though some articles were clear in their presentation and hence necessitated a “low” rating.

### 3.3. Results of Systematic Review

Table 3 provides a summary of the post and change values in lipid-lipoproteins, blood pressure, glucose, and insulin for all the 22 studies. Amongst all the studies included in Table 3, some studies had more than one intervention arm while some studies only had control group. Although no major differences in post values were observed between the control and intervention groups, change values indicate an increment in the HDL concentration coupled with a reduction in blood pressure and glucose concentration after dietary counselling.

### 3.4. Results of Meta-Analysis 

**Figure 2:**
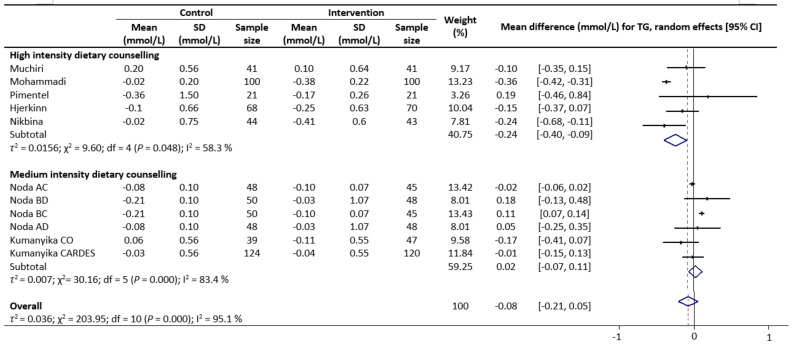
Random-effects model meta-analysis for comparing the changes in TG of RCTs providing dietary counselling compared to not providing dietary counselling and subcategory analysis in accordance with the intensity level for dietary counselling. Each study is identified by author and year. The horizontal line represents the effect size for each study and the whiskers extending on both sides represent the study effect’s 95% CI. The diamond indicates the overall effect size. Abbreviations: AC, group A and group C; BD, group B and group D; BC, group B and group D; AD, group A and group D; CO, counselling only; CARDES, components of cardiovascular dietary education system.

**Figure 3:**
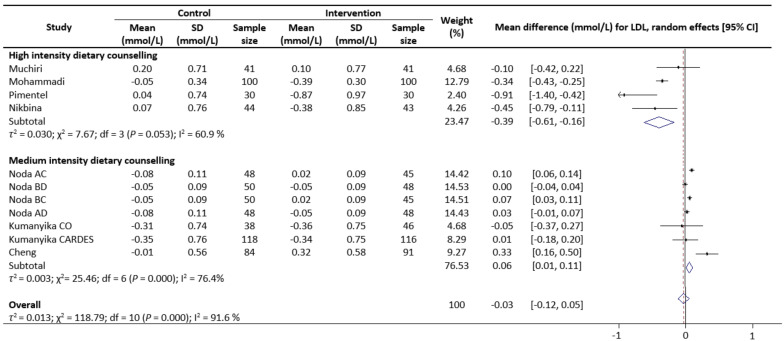
Random-effects model meta-analysis for comparing the changes in LDL of RCTs providing dietary counselling compared to not providing dietary counselling and subcategory analysis in accordance with the intensity level for dietary counselling. Each study is identified by author and year. The horizontal line represents the effect size for each study and the whiskers extending on both sides represent the study effect’s 95% CI. The diamond indicates the overall effect size. Abbreviations: AC, group A and group C; BD, group B and group D; BC, group B and group D; AD, group A and group D; CO, counselling only; CARDES, components of cardiovascular dietary education system.

**Figure 4:**
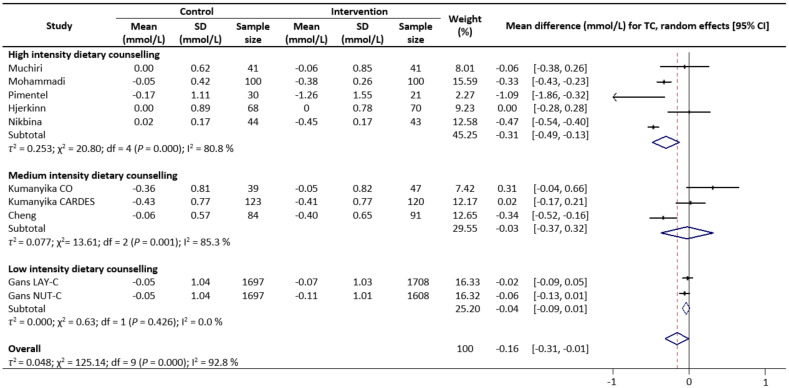
Random-effects model meta-analysis for comparing the changes in TC of RCTs providing dietary counselling compared to not providing dietary counselling and subcategory analysis in accordance with the intensity level for dietary counselling. Each study is identified by author and year. The horizontal line represents the effect size for each study and the whiskers extending on both sides represent the study effect’s 95% CI. The diamond indicates the overall effect size. Abbreviations: AC, group A and group C; BD, group B and group D; BC, group B and group D; AD, group A and group D; CO, counselling only; CARDES, components of cardiovascular dietary education system; LAY-C, lay person counselling; NUT-C, nutritionist counselling.

**Figure 5:**
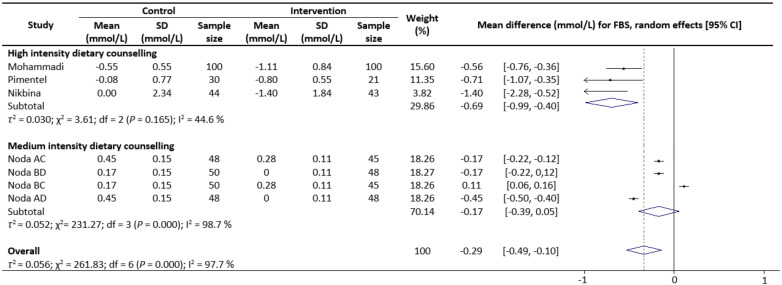
Random-effects model meta-analysis for comparing the changes in FBS of RCTs providing dietary counselling compared to not providing dietary counselling and subcategory analysis in accordance with the intensity level for dietary counselling. Each study is identified by author and year. The horizontal line represents the effect size for each study and the whiskers extending on both sides represent the study effect’s 95% CI. The diamond indicates the overall effect size. Abbreviations: AC, group A and group C; BD, group B and group D; BC, group B and group D; AD, group A and group D.

Meta-analysis results reveal that there were no significant differences in the change values of TG (WMD: −0.08 mmol/L, 95% CIs: −0.21 to 0.05) (Figure 2) and LDL (WMD: −0.03 mmol/L, 95% CIs: −0.12 to 0.05) concentrations between the control and intervention groups (Figure 3). However, dietary counselling provided favourable effect in TC (WMD: −0.16 mmol/L, 95% CIs: −0.31 to −0.01) (Figure 4) and FBS (WMD: −0.29 mmol/L, 95% CIs: −0.49 to −0.10) concentrations (Figure 5). For the analyses, the observed trends were robust and mainly stable to sensitivity analysis (Supplementary Table S2).

Sub-group analyses were conducted for TG, TC, LDL and FBS outcomes based on the intensity of the dietary counselling and the provider of dietary counselling. For the intensity sub-group analysis, TC had three categories (low, medium, and high) whilst the remaining had two categories (medium and high). High intensity of dietary counselling showed a decrease in TG (WMD: −0.24 mmol/L, 95% CIs: −0.40 to −0.09) (Figure 2), LDL (WMD: −0.39 mmol/L, 95% CIs: −0.61 to −0.16) (Figure 3), TC (WMD: −0.31 mmol/L, 95% CIs: −0.49 to −0.13) (Figure 4) and FBS (WMD: −0.69 mmol/L, 95% CIs: −0.99 to −0.40) (Figure 5) concentrations however the same impact was not reflected in the medium intensity for TG (WMD: 0.02 mmol/L, 95% CIs: −0.07 to 0.11), LDL (WMD: 0.06 mmol/L, 95% CIs: 0.01 to 0.11), TC (WMD: −0.03 mmol/L, 95% CIs: −0.37 to 0.32)and FBS (WMD: −0.17 mmol/L, 95% CIs: −0.39 to 0.05). The low intensity dietary counselling has also showed no impact on the TC (WMD: −0.04 mmol/L, 95% CIs: −0.09 to 0.01). Additionally, differences between high intensity and medium intensity in TG, LDL, and FBS were statistically significant with the non-overlap of 95% CIs. There was also no overlap of 95% CIs observed between the high and low intensity in TC.

According to the analysis for the provider of dietary counselling, involvement of dietitian showed no effects for TG (Supplementary Figure S1) and FBS (Supplementary Figure S2) concentrations while an unfavourable effect for LDL concentrations (Supplementary Figure S3). Results were similar for counselling that employed all other providers for TG and LDL concentrations, however, a significant and favourable response was observed for FBS concentrations.

## 4. Discussion

Dietary counselling has been identified as one of the beneficial nutritional strategies that improves cardiometabolic health, but its effectiveness has not been systematically studied. The findings from this systematic review and meta-analysis support evidence that dietary counselling can provide an improvement in cardiometabolic health parameters in middle-aged and older adults. Moreover, this improvement was found to be more pronounced with the provision of high intensity dietary counselling.

Due to the lack of data, meta-analysis for SBP and DBP were not able to be carried out while systematic review result demonstrated an improvement in blood pressure with dietary counselling in middle-aged and older adults. Included studies which reported blood pressure lowering responses featured a similar set of dietary counselling guidelines such as the reduction of dietary saturated fat intake and increment of dietary fibre intake. Decreasing saturated fat intake and increasing dietary fibre is in accordance with guidelines for a DASH diet and a Mediterranean diet [51,52]. With reference to a previous meta-analysis of RCTs conducted by Ndanuka et al. [53], adherence to a DASH diet and a Mediterranean diet favourably lowered systolic and diastolic blood pressure. Although the potential underlying mechanisms of dietary fibre on lowering blood pressure have not been fully elucidated, one possibility is the modulation of insulin metabolism [54,55]. Insulin may contribute to blood pressure regulation and insulin resistance and compensatory hyperinsulinemia is suggested to be a major risk factor for the development of hypertension [56]. Previous studies found increased insulin sensitivity and improved vascular endothelial function with dietary fibre consumption [57,58]. Additionally, the consumption of dietary fibre rich foods such as fruits and vegetables may contribute to the overall blood pressure lowering effect as they contain substantial amount of nitrates which increase the plasma levels of nitrate and nitrite. Those are important substrate for producing nitric oxide which is a known as vasodilator and can regulate blood pressure [59,60]. Furthermore, a positive association between saturated fat intake and blood pressure has also been reported [61] and a previous RCT reported that consumption of a meal with decrease in saturated fatty acid while increase in monounsaturated fatty acid led to a decrease in DBP [62].

Meta-analysis results revealed that when middle-aged or older adults participated in dietary counselling, they showed lowered TC and FBG concentration. A study conducted by Rijnaarts et al. showed an increase in fibre consumption after the provision of tailored dietary counselling [63]. Dietary fibre plays a pivotal role in the management of lipid metabolism and glycaemic control [64,65]. This is also supported by a meta-analysis evaluating the impact of increased fibre intake on the FBG in patients with type 2 diabetes mellitus [66]. Furthermore, previous studies have also reported a significantly lower FBG concentration when the intervention group consumed higher dietary fibre [67,68]. Several studies within our meta-analysis also revealed an increase in fibre intake and decrease in saturated fat intake after the provision of dietary counselling [23,45]. A systematic review and meta-analysis conducted to review trials of physical activity or dietary counselling to prevent cardiovascular disease found out that in high intensity counselling trials, intervention group participants showed greater increases in fruits and vegetables consumption [19]. This review also reported that high intensity trials collectively resulted in moderate to large decrease in total fat and saturated fat intake in participants of the intervention group while low and medium intensity intervention resulted in smaller reductions in fat intake which is in accordance to our findings [19]. According to the observation of an animal study, Wistar rats fed with palm (saturated) oil resulted in a lower rate of insulin stimulated glucose metabolism and insulin binding to cells when compared to sunflower (polyunsaturated) oil [69]. This result is in accordance to another animal study that a highly saturated diet decreased the rate of insulin stimulated glucose transport [70]. Therefore, the decrease in saturated fat intake may be a contributing source to the lowered FBG. The dietary counselling content may also play a role in the subsequent outcome, especially for the case in FBS where selected studies were focusing on patients with type 2 diabetes mellitus and tailored the counselling to the improvement of glycemic control in type 2 diabetes mellitus patients.

The sub-group analysis results for favourable effects on TG, TC, LDL and FBS with high intensity dietary counselling. In relevance to our study, a high intensity dietary counselling consists of six or more contacts during the intervention period and each lasting more than 30 min. Consistent with our findings, a systematic review and meta-analysis conducted by Rees et al. [71], showed that high intensity dietary intervention involving more than three scheduled personal contact is associated with reduction in TG than low intensity dietary intervention. This further supported our findings on the importance of intervention intensity and that higher intensity counselling may provide more time for adaptation to a healthy diet in order for beneficial changes to be observed consistently. The impact of duration of the study was also one of the considerations for this review, however the duration for each study varies greatly. In our review, studies that we collected results for the cardiometabolic outcomes have various length of study duration ranging from 12 weeks to 144 weeks [23,35,39,40,45]. Amongst the studies, a study conducted by Mohammadi et al. [23] with a trial length of 12 weeks has shown favourable response to TG level as compared to a study conducted by Kumanyika et al. [42] with a trial length of 48 weeks. Between the studies, Mohammadi et al. [23] reported an increase in diabetic nutrition knowledge score pre and post intervention with an indicated compliance in dietary counselling component after the provision of eight counselling sessions while Kumanyika et al. [42] reported no statistically significant difference between groups in their adherence and usage of the counselling material after the provision of four counselling sessions. This finding indicates that as compared to the duration of the study, the intensity of the dietary counselling which accounts for the length and frequency of the dietary counselling given during the duration of the study may provide a greater influence on the subject’s compliance to the program.

In contrast, the sub-group analysis results for TG, LDL and FBS did not favour counselling conducted by dietitians. This observation is in accordance to a RCT conducted by Wong et al. [18] that a dietary counselling conducted by dietitian conferred no beneficial results on blood lipids profile and blood pressure. In this RCT, counselling was only provided once in the beginning of the intervention. Under the dietitian led sub-group analysis, it consisted of two studies, Muchiri et al. [40] and Noda et al. [41], and the level of dietary counselling intensity for Muchiri et al. [40] is high while medium for and Noda et al. [41]. Whereas, for majority of the studies under All Others provided high intensity dietary counselling. A study conducted to investigate the effect of structured dietary advice provided by dietitian compared to standard advice provided by physician on dietary changes to lower the blood LDL found out that dietitians were more effective when the intensity of counselling was high with 6 counselling sessions at 30 min per session whereas the physician group only had one face-to-face consultation [72]. The study observed sustainable dietary changes and greater dietary adherence and this may be explained by the higher intensity of the dietary counselling provided which allowed for better understanding of the counselling material and also the dietitians were all trained to conduct the session in accordance to national and Mediterranean dietary guidelines [72]. Collectively, the intensity of dietary counselling appears to be one of the key factors that may determine the impact of dietary counselling on the cardiometabolic outcomes regardless of the counselling provider. Several studies [30,41,42] did not discuss the compliancy of the study subjects to the dietary counselling intervention. Whereas other studies [35,40,45] investigated and discussed the compliance of the study subjects to the intervention. Under the dietitian led group, Muchiri et al. [40] showed no significant changes in the macronutrient, fruits and vegetables intakes. Whereas for studies under All Others, Pimentel et al. [45] observed a decrease in energy, total fat, saturated fat and cholesterol intake in subjects post intervention and in case of Mohammadi et al. [23], compliance to the intervention was not studied but the knowledge scores on diabetes were significantly increased at post intervention. Compliance in nutrition education studies has an overt importance when it comes to dietetic control [73].

The key strength of this systematic review and meta-analysis is compiled data from dietary intervention studies and findings can provide causality between dietary counselling and its impact on cardiometabolic outcomes in middle-aged and older adults who show an increased risk of cardiometabolic diseases [74]. Another strength lies in its wide inclusion criteria, which shortlist all studies that provided dietary counselling without restrictions in terms of subject conditions and counselling settings. This allowed us to have a comprehensive overview of the impact of dietary counselling as a whole as compared to previous systematic review and meta-analysis which were done with reference to subjects with particular diseases, settings or specific key dietary components [75,76,77]. Exclusion of other lifestyle interventions allowed us to solely examine the impact of dietary counselling on the cardiometabolic outcomes. Lastly, this study investigated the contributing components of dietary counselling that may potentially explain the effect of dietary counselling on the cardiometabolic health via the application of subgroup analysis.

However, a few limitations of this review warrant a discussion. Assessment of adherence to the dietary counselling is important to evaluate the impact of dietary counselling, while the majority of the studies did not examine the adherence of dietary intake, or the adherence was studied via self-reported dietary intake. The quality of the studies also necessitates further discussion, only one third of the articles obtained a ‘low-risk’ bias due to the inevitable nature of dietary counselling. Subjects undergoing intervention were unable to be blinded and hence resulting in only single blinding. Additionally, the characteristic information regarding the studies which may reveal significant and meaningful insights were unreported. The meta-analytic portion of this review was limited by the significant high heterogeneity observed across studies due to the small number of studies available where the sub-group analyses utilised results from a single study with multiple arms. Besides the limited number of studies available, variation between the studies may also lead to high heterogeneity such as the wide range of dietary counselling content for each intervention and disease state of the participants. Lastly, all the trials involved in this review assessed only the classical cardiometabolic health parameters such as blood pressure and blood lipids panel without the inclusion of some other risk factors such as the body mass index, anthropometry measurement and inflammatory markers. The untenable importance of classical risk factors for cardiometabolic health are largely illustrated, however, the incorporation of other novel or clinical parameters may be helpful to improve risk prediction models and provide more insights on the cardiometabolic health status [78].

## 5. Conclusions

In conclusion, the findings from this systematic review and meta-analysis suggest that dietary counselling is a beneficial dietary strategy to improve cardiometabolic health in middle-aged and older adults with the emphasis on the counselling intensity. In particular, these findings can provide insights for dietary counselling providers to establish well-designed counselling sessions with greater consideration of the dietary counselling intensity to improve cardiometabolic health.

## Figures and Tables

**Table 1:** PICOS criteria for the inclusion of studies.

Variable	Description
Population	Adults mean aged ≥50 years old
Intervention	Groups that underwent dietary counselling
Comparator	Groups that did not go through dietary counselling
Outcome	Changes in TC, TG, HDL, LDL, FBS and insulin concentrations, SBP and DBP levels
Study Design	Randomised Controlled Trials
Research Question	Does the intensity of dietary counselling impact on the cardiometabolic health in middle-aged and older adults?

DBP, diastolic blood pressure; FBS, fasting blood glucose; HDL, high density lipoprotein; LDL, low density lipoprotein; PICOS, participant, intervention, comparison, outcome, and study design; SBP, systolic blood pressure; TC, total cholesterol; TG, total triglycerides.

**Table 2:** Characteristics of the 22 included RCTs for systematic review and meta-analysis.

First Author, Year	Country of Study, Intervention Duration (Weeks)	Population Size, Description, Mean Age (Years), BMI (kg/m^2^)	Characteristics of Intervention Group (Provider; Number of Visit; Length of Visit (Minutes); Content)	Characteristics of Control Group (Provider; Number of Visit; Length of Visit (Minutes); Content)	Dietary Compliance	Attrition Rate (%)	Outcomes Measured
Kaliora [31], 2016	Greece, 24	55, Men and women with NAFLD, 52, 29.1	Dietitian; NR; NR; Received dietary counselling on calorie restriction	Nil	Monitored via non-scheduled phone calls with 24-h dietary recall	20.0	TC, TG, LDL, HDL, SBP, DBP, FBS, Insulin
Muchiri [40], 2016	South Africa, 48	82, Men and women with type 2 diabetes, 59, 31.5	Dietitian and Research staff; 8; 120; Received educational materials and took part in nutritional education programme	Dietitian; 1; Not applicable; Received educational materials only and usual medical care.	Follow-up sessions (four monthly meetings and two bi-monthly meetings each lasting 90 min)	7.3	TC, LDL, HDL, TG, SBP DBP
Wu [33], 2013	Taiwan, 24	135, Men and women with chronic kidney disease, NR	Registered Dietitian; 6; NR; Received dietary counselling as recommended by the Kidney Disease Outcomes Quality Initiative nutritional guidelines and consumed a packet of non-protein calorie supplement	Registered Dietitian; 6; NR; Received dietary counselling as recommended by the Kidney Disease Outcomes Quality Initiative nutritional guidelines	NR	19.3	TC, TG, LDL, HDL
Al-Shookri [27], 2012	Oman, 24	100, Men and women with type 2 diabetes, 52, 28.4	Dietitian; 3; 150; Received practice guidelines nutritional care for T2DM	Dietitian; 1; 60; Received usual nutritional care	If goals and behavioural objectives of educations are not met, a second follow-up appointment would be scheduled	15.0	TC, TG, LDL, HDL, FBS
Noda [41], 2012	Japan, 4	200, Men and women with type 2 diabetes, 65, 26.2	Registered Dietitian; 4; 30–60; Face to face dietary counselling on	Nil	1 telephone counselling lasting 10–20 min in between study where the dietary performance is checked by the dietitian.	6.5	TG, HDL, LDL, SBP, DBP, FBS
Wang [34] 2012	China, 12	90, Men and women with metabolic syndrome, 51, 28	Research staff; 5; NR; Received dietary counselling based on the American Heart Association Step I diet	Nil	A random phone call once every 2 weeks was conducted to check on compliance.	4.4	TC, TG, LDL, FBS, Insulin
Klein [29], 2011	Brazil, 15	66, Men and women with type 2 diabetes and pre-diabetes, 60, 29.8	Research staff; NR; NR; Dietary counselling to improve glycaemic and lipid profiles based on the Brazilian and American Diabetes Associations guidelines	Nil	3-day food record was collected during baseline and throughout study periods.	12.1	TC, TG, LDL, HDL
Pimentel [45], 2010	Brazil, 48	67, Men and women who are at high risk of type 2 diabetes, 60, 28	Nutritionists; 36; 30–90; Individual and group counselling session including written and oral didactic instructions to improve diet quality	Nil	NR	23.9	TC, TG, LDL, HDL, FBS, Insulin
Thomson [28], 2010	United States, 24	40; overweight postmenopausal breast cancer survivors, 56, 31.8	Dietitian; 6; 45; Face to face dietary counselling for either low-fat or low-carbohydrate diet	Nil	A review of gram intake log and a 24-h recall to assess adherence to targeted diet.	0.0	TC, TG, LDL, HDL, SBP, DBP, FBS, Insulin
Gans [36], 2006	England, 24	10,144, NR, 50, 26.9	Trained lay person or Nutritionist; 1; 10–12; Dietary counselling provided according to the National Cholesterol Education Program	Received feedback tips sheet only OR Received tip sheet plus Rate Your Plate OR Received all materials along with CD audio intervention	Follow-up and assessed at baseline, 3 months, and 12 months.	NR	TC
Hjerkinn [35], 2006	Europe, 144	278, Men with high cardiovascular risks, 70, 26.5	Clinical nutritionist; 6; 30–45; Dietary counselling provided according to the European guidelines	Nil	Additional follow-up for subjects with poor adherence.	15.1	TC, TG, HDL, SBP, DBP
Schwab [44], 2006	Finland, 12	68, Men and women with type 2 diabetes, 53, 28.8	Nutritionist; 4; NR; Dietary counselling aimed to meet the generally recommended dietary goals for the prevention of cardiovascular diseases	Nil	4-day food record was kept monitoring food intake during the third and the seventh week for personal nutrition counselling.	2.9	TC, TG, LDL, HDL, SBP, DBP, FBS
Takahashi [43], 2006	Japan, 48	550, Generally healthy men and women, 56, 23.4	Research staff; 4; 15; Individual and group session with individualized dietary counselling	Research staff; 1; 15; Tailored dietary education	A second dietary assessment was performed, and the same individual dietary counselling was given to each subject.	18.5	SBP, DBP
Cheng [30], 2004	United States, 16	208, Men and women who are hypercholesterolemic, 54, 31.8	Research assistant; 4; 5–50; Received nutrition counselling using the Food For Heart Program curriculum	Nil	Every visit featured a quiz and advice sheets for each subject individually and a dietary risk assessment completed for all visits	15.9	TC, LDL, HDL
Lindgärde [26], 2001	Sweden, 54	382, Men and women with at least one of the obesity-associated CHD risk factors, 53, 33.2	Practice nurse; 8; NR; Self-help weight control educational package with leaflets and videotape	Nil	Each visit subjects have to update on their frequency of watching the video	1.6	SBP, DBP, TC, TG, LDL, HDL, FBS
Henkin [37], 2000	Israel, 48	136, Men and women with hyper cholesterol, 50, 27 136, Men and women with hyper cholesterol, 51, 26	Physicians and Dietitians; 1; 30; Counselling focused on smoking cessation, physical activity, weight control, and dietary modifications conforming to the Step I diet Physicians and Dietitians; 2–4; 30; Face to face dietary counselling session on general information about hyper- cholesterolemia, guidelines, as well as specific dietary recommendations	Nil	3-day food diary Detailed analysis of nutrient intake and eating habits guided by 3-day food diary and provision of more specific dietary recommendations.	13.2	TC, TG, LDL, HDL
Kumanyika [42], 1999	United States, 48	330, Men and women with either hypercholesterolemia or hypertension, NR, NR	Nutritionist; 4; NR; Received baseline counselling using the Cardiovascular Dietary Education System which aimed to educate about cardiovascular disease	Nutritionist; NR; NR; Received baseline counselling	Followed-up visits was given to reinforce self-directed learning.	22.7	TG, TC, LDL, HDL
Rodríguez-Morán [46], 1998	Mexico, 12	60, Men and women with type 2 diabetes, 57, 28.6	Research staff; NR; NR; NR	Nil	NR	0.0	TC, TG, LDL, HDL
Samuelsson [32], 1997	Sweden, 48	28, Men and women with non-diabetic primary renal disease and moderately advanced renal insufficiency, 50, 26.9	Dietitian; NR; NR; Received tailored dietary counselling for diabetes	Nil	Telephone follow-up after 1 month	10.7	TC, TG, LDL, HDL, vLDL
Nikbina [39], 2020	Iran, 16	90, Men and women with type 2 diabetes, 52, NR	Nutritionist; 5; 45–90; Educational content included healthy nutrition that focused on balance, diversity and diet, proper food replacement technique, how to use the healthy eating plate and cook healthy food	Received only routine care	Telephone follow-up over a period of 4 months on a weekly basis conducted by a nutritionist	3.3	SBP, DBP, TC, TG, LDL, HDL, FBS
Mohammadi [23], 2018	Iran, 12	240, Men and women with type 2 diabetes, 51, 27	Researcher; 8; 120; Received dietary counselling and education booklet, complications, self-care and self-efficacy behaviour, physical activity, healthy diet, medication adherence and to self-monitor their blood glucose level	Researcher; NR; NR; Received conventional dietary counselling with no additional details	NR	16.7	TC, TG, LDL, HDL, FBS
Meuleman [38], 2017	Netherlands, 24	151, men and women with moderately decreased kidney functions, 55; 29.7	Dietitian; 2; 60; Received regular care according to the Dutch Federation of Nephrology treatment guidelines and self-management intervention with personal coaches including psychologists	Received only regular care according to the Dutch Federation of Nephrology treatment guidelines	Feedbacks via telephone from coach to discuss progression, achievements, barriers, and possible solutions	8.6	SBP, DBP

CHD, Chronic heart disease; DBP, diastolic blood pressure; FBS, fasting blood glucose; HDL, high density lipoprotein; LDL, low density lipoprotein; NAFLD, Non-alcoholic fatty liver disease; NR, Not reported; SBP, systolic blood pressure; TC, total cholesterol; TG, total triglycerides; vLDL, Very-low density lipoprotein.

**Table 3:** Summary of post and change values in lipid-lipoproteins, blood pressure, glucose, and insulin of all the 22 studies.

Outcome (Unit)	Post		Change	
	Control	Intervention	Control	Intervention
TC (mmol/L)	5.25	5.41	−0.10	−0.19
	[4.23, 5.94]	[4.25, 6.24]	[−0.36, 0.02]	[−1.26, 0.17]
TG (mmol/L)	1.76	1.85	−0.10	−0.11
	[1.49, 2.16]	[0.95, 4.76]	[−0.42, 0.20]	[−0.41, 0.18]
LDL (mmol/L)	3.16	3.34	−0.12	−0.15
	[2.65, 4.09]	[2.49, 4.62]	[−0.36, 0.07]	[−0.88, 0.37]
HDL (mmol/L)	1.25	1.31	−0.01	0.06
	[1.02, 1.44]	[0.91, 1.63]	[−0.09 0.10]	[−0.11, 0.20]
SBP (mmHg)	136.7	132.0	−0.10	−4.86
	[128.5, 143]	[121.8, 142]	[−5.00, 4.00]	[−11.80, −0.80]
DBP (mmHg)	81.0	78.9	−0.94	−2.94
	[75.9, 89.0]	[74.8, 85.1]	[−4.00, 1.00]	[−7.50, 1.60]
Glucose (mmol/L)	7.00	6.76	−0.02	−0.39
	[5.05, 9.17]	[5.08, 11.90]	[−0.08, 0.02]	[−2.05, 0.32]
Insulin (μU/mL) ^a^	14.5	14.6	−1.10	−1.43
	-	[11.40, 18.70]	-	[−4.50, 1.93]

Data represented as mean and range. C, Control; DBP, Diastolic blood pressure; HDL, High-density lipoprotein; I, intervention; LDL, Low-density lipoprotein; SBP, Systolic blood pressure; TC, Total cholesterol; TG, Triglycerides; ^a^ Range values of insulin for control group were not reported as there was only one study. TC (Intervention [23,26,27,29,30,31,32,33,34,35,36,37,39,40,42,44,45,46,50], Control [23,30,33,35,36,37,40,42,45]); TG (Intervention [23,26,27,29,31,32,34,35,37,39,40,41,42,44,45,46,50], Control [35,40,41,42,45]); LDL (Intervention [23,26,27,28,29,30,31,32,34,37,39,40,41,42,44,45,46], Control [30,33,40,41,42,45]). HDL (Intervention [23,26,27,29,30,31,32,35,37,39,40,41,42,44,45,46,50], Control [30,33,35,40,41,42,45]); SBP (Intervention [26,28,31,35,38,40,41,43,44], Control [35,38,39,40,41,43]); DBP (Intervention [26,28,31,35,38,40,41,43,44], Control [35,38,39,40,41,43]); Glucose (Intervention [23,26,27,28,31,34,41,44,45], Control [39,41,45]; Insulin (Intervention [28,31,34,45], Control [45].

## Data Availability

The data presented in this study will be made available on request from the corresponding author.

## Supplementary Materials

The following are available online at https://www.mdpi.com/article/10.3390/nu13092936/s1, Figure S1: Random-effects model meta-analysis for comparing the changes in TG of RCTs providing dietary counselling compared to not providing dietary counselling and subcategory analysis in accordance with the provider of dietary counselling. Each study is identified by author and year. The horizontal line represents the effect size for each study and the whiskers extending on both sides represent the study effect’s 95% CI. The diamond indicates the overall effect size. Abbreviations: AC, group A and group C; BD, group B and group D; BC, group B and group D; AD, group A and group D; CO, counselling only; CARDES, components of cardiovascular dietary education system. Figure S2: Random-effects model meta-analysis for comparing the changes in FBS of RCTs providing dietary counselling compared to not providing dietary counselling and subcategory analysis in accordance with the provider of dietary counselling. Each study is identified by author and year. The horizontal line represents the effect size for each study and the whiskers extending on both sides represent the study effect’s 95% CI. The diamond indicates the overall effect size. Abbreviations: AC, group A and group C; BD, group B and group D; BC, group B and group D; AD, group A and group D. Figure S3: Random-effects model meta-analysis for comparing the changes in LDL of RCTs providing dietary counselling compared to not providing dietary counselling and subcategory analysis in accordance with the provider of dietary counselling. Each study is identified by author and year. The horizontal line represents the effect size for each study and the whiskers extending on both sides represent the study effect’s 95% CI. The diamond indicates the overall effect size. Abbreviations: AC, group A and group C; BD, group B and group D; BC, group B and group D; AD, group A and group D; CO, counselling only; CARDES, components of cardiovascular dietary education system. Table S1: Risk of bias assessment using Cochrane’s Modified Tool, Table S2: Sensitivity analysis following the removal of single groups or randomized controlled trials to assess the robustness of meta-analyses results studying the impact of dietary counselling fruit on cardiovascular disease risk factors in older adults.
