# Supplementary material for: A Systematic Review and Meta-Analysis of the Impact of Different Intensity of Dietary Counselling on Cardiometabolic Health in Middle-Aged and Older Adults
Source: Nutrients. 2021 Aug 25;13(9):2936. doi: 10.3390/nu13092936 (PMC8469488; doi:10.3390/nu13092936)
Supplement: Supplementary file 1 [file nutrients-13-02936-s001.zip › Nutrients_Supplementary Figures V1.pdf]

Study	Control			Intervention			Weight (%)	Mean difference (mmol/L) for TG, random effects [95% CI]	
	Mean (mmol/L)	SD (mmol/L)	Sample size	Mean (mmol/L)	SD (mmol/L)	Sample size			
**Dietitian Involvement**									
Muchiri	0.2	0.56	41	0.1	0.60	41	9.17	-0.10	[-0.35, 0.15]
Noda AC	-0.08	0.10	48	-0.1	0.07	45	13.42	-0.02	[-0.06, 0.02]
Noda BD	-0.21	0.10	50	-0.03	1.07	48	8.01	0.18	[-0.13, 0.48]
Noda BC	-0.21	0.10	50	-0.1	0.07	45	13.42	0.11	[0.07, 0.14]
Noda AD	-0.08	0.10	48	-0.03	1.07	48	8.01	0.05	[-0.25, 0.35]
Subtotal							47.98	0.04	[-0.06, 0.14]
$\tau^2 = 0.007$; $\chi^2 = 27.69$; df = 4 ($P = 0.000$); $I^2 = 85.6$ %									
**All Others**									
Mohammadi	-0.02	0.19	100	-0.38	0.22	100	13.23	-0.36	[-0.42, -0.30]
Pimentel	-0.36	1.50	21	-0.17	0.26	21	3.26	0.19	[-0.46, 0.84]
Hjerkinn	-0.1	0.66	68	-0.25	0.63	70	10.04	-0.15	[-0.37, 0.07]
Kumanyika CO	0.06	0.56	39	-0.11	0.55	47	9.58	-0.17	[-0.41, 0.07]
Kumanyika CARDES	-0.03	0.56	124	-0.04	0.55	120	11.84	-0.01	[-0.15, 0.13]
Nikbina	-0.02	0.75	44	-0.41	0.6	43	7.81	-0.39	[-0.68, -0.11]
Subtotal							52.02	-0.19	[-0.37, -0.02]
$\tau^2 = 0.033$; $\chi^2 = 26.73$; df = 5 ($P = 0.000$); $I^2 = 81.3$ %									
**Overall**							100	-0.08	[-0.21, 0.05]
$\tau^2 = 0.036$; $\chi^2 = 203.95$; df = 10 ($P = 0.000$); $I^2 = 95.1$ %									

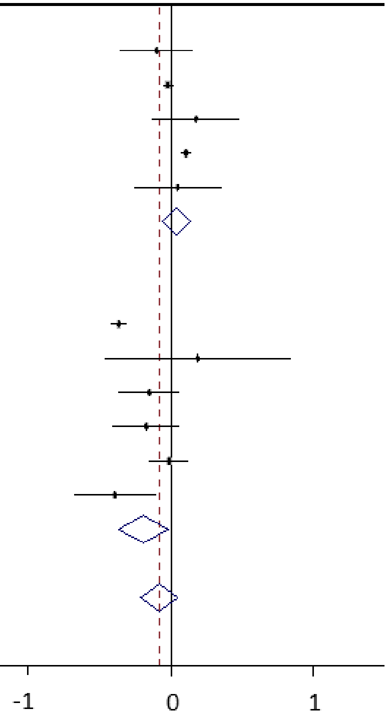

Supplementary Figure S1. Random-effects model meta-analysis for comparing the changes in TG of RCTs providing dietary counselling compared to not providing dietary counselling and subcategory analysis in accordance with the provider of dietary counselling. Each study is identified by author and year. The horizontal line represents the effect size for each study and the whiskers extending on both sides represent the study effect's 95% CI. The diamond indicates the overall effect size. Abbreviations: AC, group A and group C; BD, group B and group D; BC, group B and group D; AD, group A and group D; CO, counselling only; CARDES, components of cardiovascular dietary education system.

Study	Control			Intervention			Weight (%)	Mean difference (mmol/L) for FBS, random effects [95% CI]	
	Mean (mmol/L)	SD (mmol/L)	Sample size	Mean (mmol/L)	SD (mmol/L)	Sample size			
**Dietitian Involvement**									
Noda AC	-0.08	0.10	48	-0.1	0.07	45	18.26	-0.17	[-0.22, -0.12]
Noda BD	-0.21	0.10	50	-0.03	1.07	48	18.27	-0.17	[-0.22, -0.12]
Noda BC	-0.21	0.10	50	-0.1	0.07	45	18.26	0.11	[0.06, 0.16]
Noda AD	-0.08	0.10	48	-0.03	1.07	48	18.26	-0.45	[-0.50, -0.40]
Subtotal							70.14	-0.17	[-0.39, 0.05]
$\tau^2 = 0.052$; $\chi^2 = 231.27$; df = 3 ($P = 0.000$); $I^2 = 98.7$ %									
**All Others**									
Mohammadi	-0.02	0.19	100	-0.38	0.22	100	15.60	-0.56	[-0.76, -0.36]
Pimentel	-0.36	1.50	21	-0.17	0.26	21	11.35	-0.71	[-1.07, -0.35]
Nikbina	0.00	2.34	44	-1.40	1.84	43	3.84	-1.40	[-2.28, -0.52]
Subtotal							26.86	-0.69	[-0.99, -0.40]
$\tau^2 = 0.030$; $\chi^2 = 3.61$; df = 2 ($P = 0.165$); $I^2 = 44.6$ %									
**Overall**							100	-0.34	[-0.53, -0.14]
$\tau^2 = 0.056$; $\chi^2 = 261.83$; df = 6 ($P = 0.000$); $I^2 = 97.7$ %									

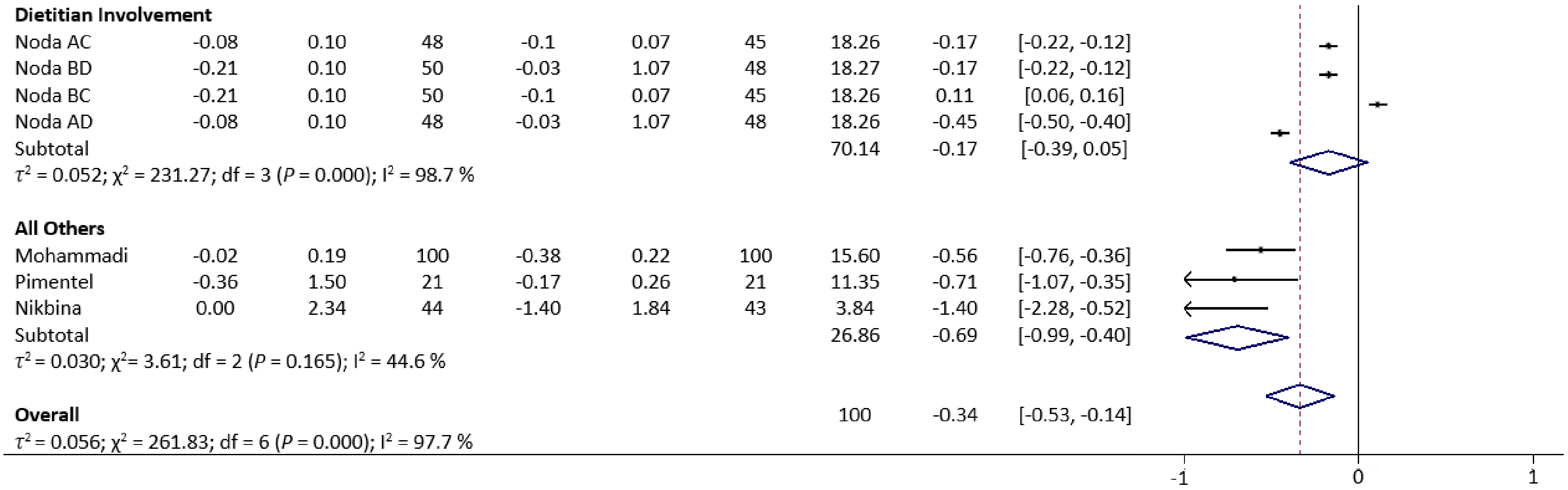

Supplementary Figure S2. Random-effects model meta-analysis for comparing the changes in FBS of RCTs providing dietary counselling compared to not providing dietary counselling and subcategory analysis in accordance with the provider of dietary counselling. Each study is identified by author and year. The horizontal line represents the effect size for each study and the whiskers extending on both sides represent the study effect's 95% CI. The diamond indicates the overall effect size. Abbreviations: AC, group A and group C; BD, group B and group D; BC, group B and group D; AD, group A and group D.

Study	Control			Intervention			Weight (%)	Mean difference (mmol/L) for LDL, random effects [95% CI]	
	Mean (mmol/L)	SD (mmol/L)	Sample size	Mean (mmol/L)	SD (mmol/L)	Sample size			
**Dietitian Involvement**									
Muchiri	0.2	0.56	41	0.1	0.60	41	4.68	-0.01	[-0.42, 0.22]
Noda AC	-0.08	0.10	48	-0.1	0.07	45	14.42	0.10	[0.06, 0.14]
Noda BD	-0.21	0.10	50	-0.03	1.07	48	14.53	0.00	[-0.04, 0.04]
Noda BC	-0.21	0.10	50	-0.1	0.07	45	14.51	0.07	[0.03, 0.11]
Noda AD	-0.08	0.10	48	-0.03	1.07	48	14.43	0.03	[-0.01, 0.07]
Subtotal							59.51	0.05	[0.01, 0.09]
$\tau^2 = 0.002$; $\chi^2 = 15.24$; df = 4 ($P = 0.004$); $I^2 = 73.8$ %									
**All Others**									
Mohammadi	-0.02	0.19	100	-0.38	0.22	100	12.79	-0.34	[-0.43, -0.25]
Pimentel	-0.36	1.50	21	-0.17	0.26	21	2.40	-0.91	[-1.40, -0.42]
Kumanyika CO	0.06	0.56	39	-0.11	0.55	47	4.68	-0.05	[-0.37, 0.27]
Kumanyika CARDES	-0.03	0.56	124	-0.04	0.55	120	8.29	0.01	[-0.18, 0.20]
Cheng	-0.01	0.56	84	0.32	0.58	91	9.27	0.33	[-0.16, 0.50]
Nikbina	0.07	0.76	44	-0.38	0.85	43	4.26	-0.45	[-0.79, -0.11]
Subtotal							40.49	-0.20	[-0.50, 0.10]
$\tau^2 = 0.120$; $\chi^2 = 62.43$; df = 5 ($P = 0.000$); $I^2 = 92.0$ %									
**Overall**							100	-0.03	[-0.12, 0.05]
$\tau^2 = 0.013$; $\chi^2 = 118.79$; df = 10 ($P = 0.000$); $I^2 = 91.6$ %									

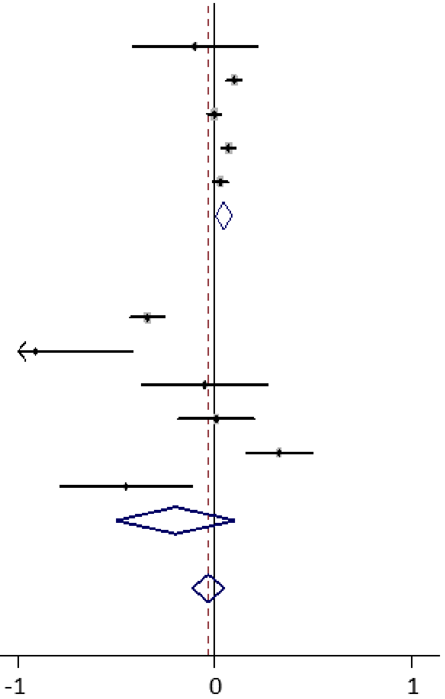

Supplementary Figure S3. Random-effects model meta-analysis for comparing the changes in LDL of RCTs providing dietary counselling compared to not providing dietary counselling and subcategory analysis in accordance with the provider of dietary counselling. Each study is identified by author and year. The horizontal line represents the effect size for each study and the whiskers extending on both sides represent the study effect's 95% CI. The diamond indicates the overall effect size. Abbreviations: AC, group A and group C; BD, group B and group D; BC, group B and group D; AD, group A and group D; CO, counselling only; CARDES, components of cardiovascular dietary education system.
