# Supplementary material for: A Systematic Review and Meta-Analysis of the Impact of Different Intensity of Dietary Counselling on Cardiometabolic Health in Middle-Aged and Older Adults
Source: Nutrients. 2021 Aug 25;13(9):2936. doi: 10.3390/nu13092936 (PMC8469488; doi:10.3390/nu13092936)
Supplement: Supplementary file 1 [file nutrients-13-02936-s001.zip › Nutrients_Supplementary table.pdf]

Supplementary Table S1. Risk of bias assessment using Cochrane's Modified Tool

Article, year	Selection bias		Other bias	Performance bias	Detection bias	Attrition bias
	Random sequence allocation	Allocation concealment	Other sources of bias	Blinding of participants and investigator	Blinding of investigator	Incomplete outcome data
Lindgärde, 2001	Unclear	Unclear	Unclear	Low	Low	Low
Henkin, 2000	Low	Unclear	Unclear	High	Unclear	Unclear
Muchiri, 2016	Low	Low	Unclear	High	High	Low
Britton, 2019	Low	Unclear	Unclear	Unclear	High	Unclear
Noda 2012	Low	Unclear	Unclear	High	Low	Unclear
Kumanyika, 1999	Unclear	Unclear	Unclear	Unclear	Unclear	Unclear
Takahashi 2006	Low	Unclear	Unclear	High	Unclear	Unclear
Tan 2016	Low	Low	Unclear	High	Unclear	Low
Schwab 2006	Unclear	Unclear	Unclear	Low	Low	Unclear
Mohammadi, 2018	Unclear	Unclear	Unclear	Unclear	Unclear	Low
Pimentel, 2010	Unclear	Unclear	Unclear	High	Unclear	Unclear
Rodríguez-Morán, 1998	Unclear	Unclear	Unclear	Low	Low	Low
Al-Shookri, 2012	Unclear	Unclear	Unclear	Unclear	Unclear	Unclear
Thomson, 2010	Unclear	Unclear	Unclear	Unclear	Unclear	Unclear
Klein, 2011	Unclear	Unclear	Unclear	High	Low	Unclear
Cheng, 2004	Low	Unclear	Unclear	High	High	Unclear
Kaliora, 2016	Low	Low	Unclear	Unclear	Low	Low
Samuelsson, 1997	Unclear	Unclear	Unclear	High	High	Unclear
Wu, 2013	Unclear	Low	Unclear	High	High	Unclear
Wang, 2012	Unclear	Unclear	Unclear	Unclear	Unclear	Unclear
Hjerkinn, 2006	Unclear	Unclear	Unclear	Low	Unclear	Unclear
Gans, 2006	High	Unclear	Unclear	Unclear	Unclear	Unclear
Nikbina, 2020	Unclear	Unclear	Unclear	Unclear	Unclear	Low

Supplementary Table S2. Sensitivity analysis following the removal of single groups or randomized controlled trials to assess the robustness of meta-analyses results studying the impact of dietary counselling fruit on cardiovascular disease risk factors in older adults.

Group excluded	Weighted mean difference	[95% CI]	I^2 (%)	χ^2	*P* for χ^2	Model
**Total Triglycerides (mmol/L)**						
None	-0.022	[-0.044, -0.001]	95.10	203.95	0.000	Random
Muchiri	-0.022	[-0.044, -0.000]	95.60	203.59	0.000	Random
Noda	-0.024	[-0.052, 0.004]	95.60	203.92	0.000	Random
Noda	-0.023	[-0.045, -0.002]	95.60	202.30	0.000	Random
Noda	-0.110	[-0.138, -0.081]	92.00	112.23	0.000	Random
Noda	-0.023	[-0.045, -0.001]	95.60	203.73	0.000	Random
Mohammadi	0.036	[0.012, 0.059]	79.10	43.14	0.000	Random
Pimentel	-0.023	[-0.044, -0.001]	95.60	203.54	0.000	Random
Hjerkinn	-0.021	[-0.043, 0.001]	95.60	202.60	0.000	Random
Kumanyika	-0.021	[-0.043, 0.001]	95.60	202.43	0.000	Random
Kumanyika	-0.023	[-0.045, -0.001]	95.60	203.92	0.000	Random
Nikbina	-0.020	[-0.042, 0.001]	95.40	197.53	0.000	Random
**Total Cholesterol (mmol/L)**						
None	-0.190	[-0.225, -0.154]	92.80	125.14	0.000	Random
Muchiri	-0.191	[-0.227, -0.156]	85.60	48.50	0.000	Random
Mohammadi	-0.168	[-0.206, -0.130]	93.10	115.81	0.000	Random
Pimentel	-0.188	[-0.223, -0.152]	93.30	119.89	0.000	Random
Hjerkinn	-0.193	[-0.228, -0.157]	93.50	123.35	0.000	Random
Kumanyika	-0.195	[-0.230, -0.159]	93.20	117.04	0.000	Random
Kumanyika	-0.197	[-0.233, -0.161]	93.40	120.49	0.000	Random
Cheng	-0.184	[-0.220, -0.148]	93.50	122.37	0.000	Random
Gan	-0.249	[-0.290, -0.208]	91.50	94.24	0.000	Random
Gan	-0.234	[-0.275, -0.193]	92.60	107.43	0.000	Random
Nikbina	-0.101	[-0.141, -0.060]	83.50	48.56	0.000	Random
**LDL-cholesterol (mmol/L)**						
None	0.030	[0.011, 0.049]	91.60	118.79	0.000	Random
Muchiri	0.030	[0.011, 0.049]	92.40	118.16	0.000	Random
Noda	0.012	[-0.009, 0.033]	91.40	105.12	0.000	Random
Noda	0.040	[0.018, 0.062]	92.20	115.46	0.000	Random
Noda	0.017	[-0.005, 0.038]	92.00	113.01	0.000	Random
Noda	0.030	[0.009, 0.051]	92.40	111.04	0.000	Random
Mohammadi	0.047	[0.028, 0.067]	81.70	49.13	0.000	Random
Pimentel	0.031	[0.012, 0.050]	91.40	104.86	0.000	Random
Kumanyika	0.030	[0.011, 0.049]	92.40	118.55	0.000	Random
Kumanyika	0.030	[0.011, 0.049]	92.40	118.55	0.000	Random
Cheng	0.026	[0.007, 0.045]	91.60	106.61	0.000	Random
Nikbina	0.031	[0.012, 0.050]	91.90	111.05	0.000	Random
**Fasting Blood Glucose (mmol/L)**						
None	-0.280	[-0.205, -0.155]	97.70	261.83	0.000	Random
Noda	-0.183	[-0.212. -0.154]	98.10	261.64	0.000	Random
Noda	-0.183	[-0.212, -0.154]	98.10	261.63	0.000	Random
Noda	-0.274	[-0.303, -0.245]	94.90	97.50	0.000	Random
Noda	-0.093	[-0.122, -0.064]	95.80	119.65	0.000	Random
Mohammadi	-0.173	[-0.199, -0.148]	98.00	247.25	0.000	Random
Pimentel	-0.177	[-0.202, -0.152]	98.00	253.53	0.000	Random
Nikbina	-0.179	[-0.204, -0.154]	98.00	254.45	0.000	Random
